# A Case Report of a Double-Lumen Cystic Duct During Cholecystectomy and a Review of the Biliary Tree Variations

**DOI:** 10.7759/cureus.115419

**Published:** 2026-08-29

**Authors:** Jorge Javier Garza-Castillo, Karen Paola Navarro Baez, José Carlos Hidalgo Cazares, Luis Zorrilla

**Affiliations:** 1 General Surgery, Dr. José Eleuterio González University Hospital, Autonomous University of Nuevo León, Monterrey, MEX; 2 General Surgery, Universidad del Valle de México, Monterrey, MEX; 3 Radiology, Dr. José Eleuterio González University Hospital, Autonomous University of Nuevo León, Monterrey, MEX

**Keywords:** bile duct variation, cystic duct anatomical variations‎, double cystic duct, double lumen cystic duct, laparoscopic cholecystectomy

## Abstract

Cholecystectomy is one of the most common elective abdominal surgeries worldwide. Although the procedure is uncomplicated in most cases, variations in the anatomy of the biliary tree can be encountered. An 18-year-old female, 30 days postpartum, presents with abdominal pain in the right hypochondrium accompanied by nausea and vomiting. Murphy's sign was negative. An upper abdominal ultrasound was performed, reporting a 6.3 x 2.1 cm gallbladder with 0.6 cm wall thickening, a well-defined hyperechoic image casting an acoustic shadow, and a 0.5 cm common bile duct. A laparoscopic cholecystectomy was performed, during which a "critical view of safety" was successfully achieved; however, upon transecting the cystic duct, a double-lumen duct was observed. Knowledge of anatomical variations of the biliary tract, whether common or rare, is vital for safety during laparoscopic cholecystectomy.

## Introduction

Cholecystectomy is one of the most common elective abdominal surgeries worldwide. Since the early 1990s, the laparoscopic approach has become the gold standard, offering superior visualization of relevant anatomical structures owing to the magnification and resolution capabilities of modern video-laparoscopic systems; consequently, the volume of these operations has risen globally. Laparoscopic cholecystectomy is associated with a higher risk of bile duct injury compared to open cholecystectomy, with reported injury rates of 0.3%-0.7% [1].

Although the procedure is uncomplicated in most cases, anatomical studies indicate that variations in the biliary tree occur in 24-47% of the population [2,3]. Abnormal anatomy of the biliary tree predisposes patients to higher risks of ductal injury and postoperative complications [4]. This case report aims to describe a new variant found during a cholecystectomy and the potential anatomical variants of the biliary tract, the clinical course, and the therapeutic management of a patient presenting with an unusual manifestation of this condition.

## Case presentation

An 18-year-old female with no significant past medical history, currently 30 days postpartum following her first pregnancy, presents with a 12-hour history of severe, colicky abdominal pain localized to the right hypochondrium and radiating to the back in a band-like distribution. The pain was accompanied by nausea and multiple episodes of vomiting; treatment with ketorolac provided no relief. She denied dysuria, vaginal discharge, or fever. Physical examination revealed mild tenderness in the right hypochondrium; Murphy's sign and Giordano's sign were negative. Laboratory results are shown in Table 1.

**Table 1:** Laboratory results Hgb: hemoglobin; WBC: white blood count; Plt: platelets; Glu: serum glucose; BUN: blood urea nitrogen; Cr: creatinine; AST: aspartate aminotransferase; ALT: alanine aminotransferase; ALP: alkaline phosphatase; AMY: amylase; BT: total bilirubin; BD: direct bilirubin; BI: indirect bilirubin; CRP: c-reactive protein

Parameters	Results	Reference Values
Hgb	10.2 g/dL	12.00-15.70 g/dL
WBC	9.47 K/uL	4.1-11.10 K/uL
Plt	222 K/uL	142-424 K/uL
Glu	93 mg/dL	60-100 mg/dL
BUN	13 mg/dL	7-20 mg/dL
Cr	0.7 mg/dL	0.4-1.0 mg/dL
AST	106 UI/L	11-32 UI/L
ALT	136 UI/L	7-45 UI/L
ALP	483 UI/L	37-104 UI/L
AMY	25 U/L	22-80 U/L
BT	1.1 mg/dL	0.2-1 mg/dL
BD	0.7 mg/dL	0.0-0.2 mg/dL
BI	0.4 mg/dL	0.2-0.8 mg/dL
CRP	0.2 mg/dL	0.0-1.0 mg/dL

An upper abdominal ultrasound was performed, reporting a 6.3 x 2.1 cm gallbladder with 0.6 cm wall thickening, a well-defined hyperechoic image casting an acoustic shadow, and a 0.5 cm common bile duct. Due to abnormal liver function tests, magnetic resonance cholangiopancreatography (MRCP) was ordered, and choledocholithiasis was ruled out (Figure 1).

**Figure 1:**
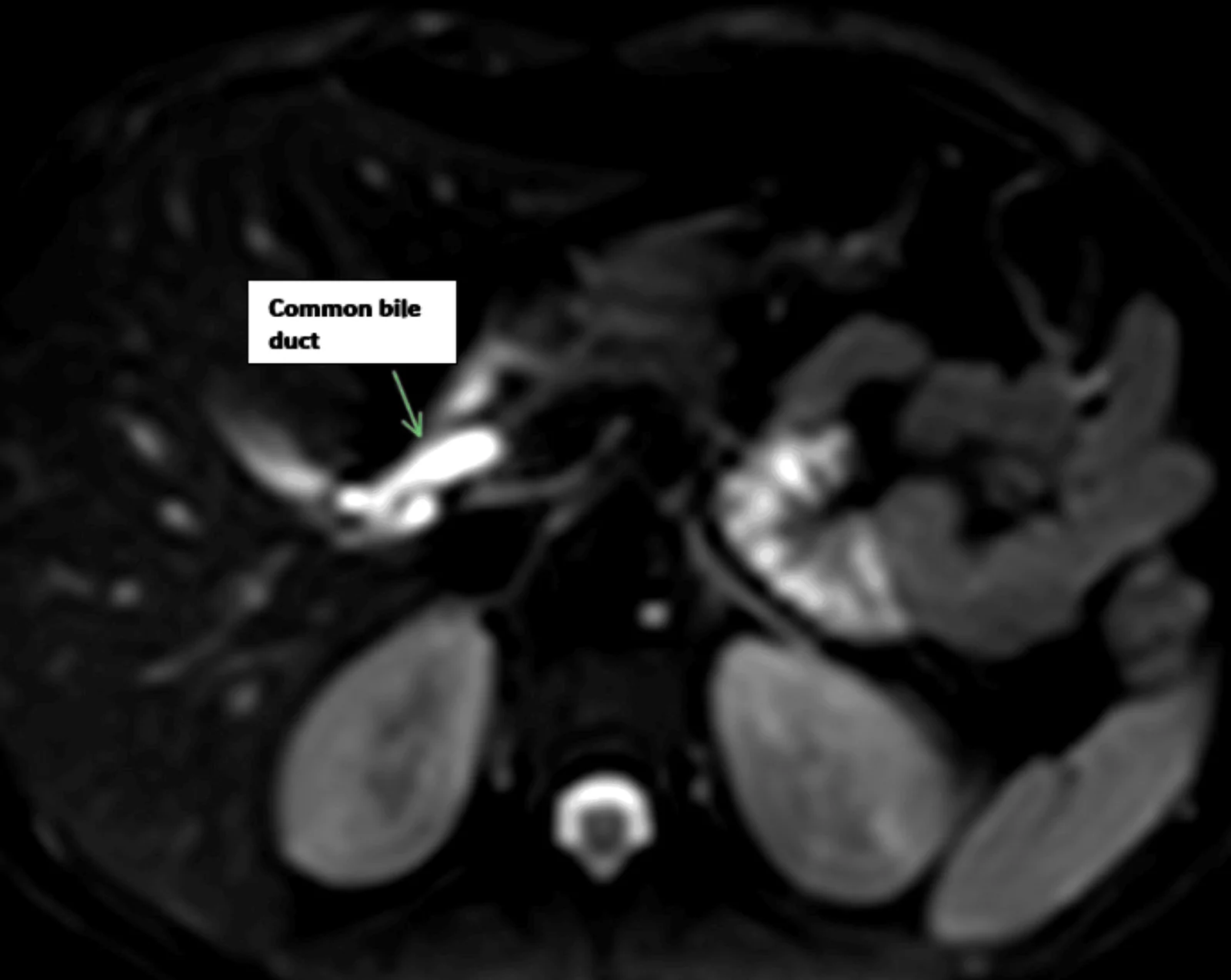
MRI showing the common bile duct without filling defects

A laparoscopic cholecystectomy was performed, during which a "critical view of safety" was successfully achieved. We found a 3-cm-long cystic duct that was in an inferior and lateral relation to the cystic artery. However, upon transecting the cystic duct, we found a duct formed by two independent channels or lumina in close contact with each other, covered by a single sheath; both having a lumen of approximately 2 mm (Video 1). The surgical site was re-explored, revealing no injury to the common bile duct or any aberrant bile duct. The surgery proceeded without evidence of bile leakage from the surgical bed, and the gallbladder was successfully resected without major complications. 

**Video 1:** Laparoscopic cholecystectomy with a double-lumen cystic duct The double-lumen duct found after division of the cystic duct with scissors during laparoscopic cholecystectomy.

The cystic duct was retrospectively reviewed on magnetic resonance imaging, and no anatomical variations could be found (Figure 2). This could be explained by the two ducts running in parallel with a diameter of 2 mm, and by an MRI slice thickness of 3-4 mm.

**Figure 2:**
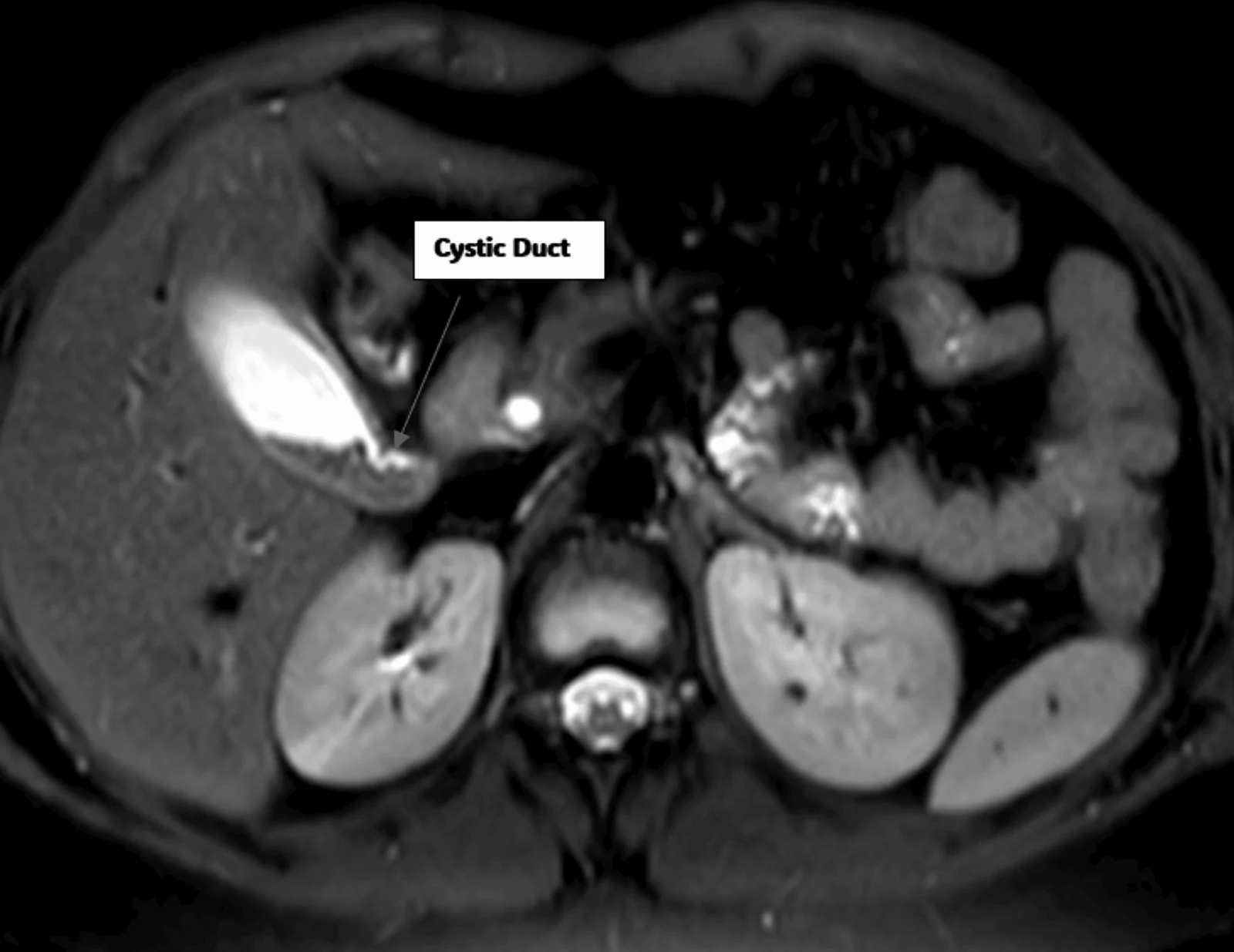
MRI showing the cystic duct without evidence of an anatomical variation at this portion of the billiary tree

Histopathological analysis revealed a gallbladder measuring 8.5 x 3.3 x 1.2 cm. Upon sectioning, the wall measured 0.2 cm in thickness, and the mucosa appeared velvety, light brown with yellowish streaks, with multiple gallstones. The pathological diagnosis was chronic cholecystitis with cholelithiasis and cholesterolosis.

The patient was discharged on postoperative Day 3 following laboratory testing, which showed a decrease in liver function tests, bilirubin, and leukocyte counts (Table 2). She returned for a follow-up visit 12 days after discharge; she remained clinically stable, and repeat laboratory tests showed no abnormalities.

**Table 2:** Postoperative laboratory results Hgb: hemoglobin; WBC: white blood count; AST: aspartate aminotransferase; ALT: alanine aminotransferase; ALP: alkaline phosphatase; BT: total bilirubin; BD: direct bilirubin; BI: indirect bilirubin

Parameter	Results	Reference Values
Hgb	9.8 g/dL	12.00-15.70 g/dL
WBC	6.32 K/uL	4.1-11.10 K/uL
AST	17 UI/L	11-32 UI/L
ALT	45 UI/L	7-45 UI/L
ALP	233 UI/L	37-104 UI/L
BT	0.3 mg/dL	0.2-1 mg/dL
BD	0.1 mg/dL	0.0-0.2 mg/dL
BI	0.2 mg/dL	0.2-0.8 mg/dL

## Discussion

The presence of anatomical variations during laparoscopic cholecystectomy remains a major challenge for the surgeon. Intra- and extrahepatic biliary tract variants are not uncommon. A classic anatomic picture between the extrahepatic bile duct, cystic duct, and related arteries is seen in only 33% of individuals [5].

To illustrate these variations that affect the safety of cholecystectomy, we can use the classifications proposed by Janssen et al. and Kurahashi et al. [6,7]. Regarding the former, types 4 and 5 are the most relevant for gallbladder surgery. Type 4 was found in 6.3% of patients and type 5 in 0.6% [6]. Both carry a high risk of bile duct injury due to the relationship between the posterior right duct and the cystic duct. Regarding the latter, a study was conducted in which cases were subclassified into high- and low-risk categories; it was found that laparoscopic subtotal cholecystectomy was performed more frequently in the high-risk group than in the low-risk group [7]. In this patient, there was no aberrant right hepatic duct joining the cystic duct.

The gallbladder and cystic duct also exhibit multiple anatomical variations. Findings range from double gallbladders with independent or fused ducts to septated gallbladders or those with diverticula. Uncommon cystic duct variants include ducts that bifurcate before joining the common hepatic duct or ducts that connect to the right intrahepatic branch [8].

The most important variable to consider during surgery is the junction point where the cystic duct meets the common hepatic duct. An angular junction is the most frequent, found in 75% of patients. The cystic duct may run parallel to the common hepatic duct in 20% of cases, while in 5%, the junction may follow a spiral course [6,8].

The presence of a double-lumen cystic duct is extremely rare. Otaibi et al. stated that a double cystic duct is associated with a double gallbladder in 80% of cases [9]. Meanwhile, Chrysikos et al. found a double cystic duct in 0.38% of the population [10]. Preoperatively, it is difficult to observe this variation in imaging studies, but the use of indocyanine green transoperatively could potentially identify this rare variation.

## Conclusions

A double-lumen cystic duct is a unique anatomical variant with very limited information in the literature. The discovery of this variant during cholecystectomy is extremely rare and should prompt a reassessment of the case with the intention of documenting and reporting this finding.

Knowledge of anatomical variations of the biliary tract, whether common or rare, is vital for safety during laparoscopic cholecystectomy. Identifying these variations in preoperative imaging studies, when possible, can aid in the procedure's performance, without forgetting other safety measures, such as the use of indocyanine green during surgery.

## References

[1] Triantafyllou G, Gondorf D, Manatakis DK (2026). Intraoperative variations of cystic artery and duct in laparoscopic cholecystectomy: a systematic review with meta-analysis. ANZ J Surg.

[2] Munie S, Nasser H, Go PH, Rosso K, Woodward A (2019). Case report of a duplicated cystic duct: a unique challenge for the laparoscopic surgeon. Int J Surg Case Rep.

[3] Aguirre-Olmedo Aguirre-Olmedo, Fernández-Castro Fernández-Castro, González-Angulo Rocha, Cárdenas-Lailson Cárdenas-Lailson, Beristain-Hernández Beristain-Hernández (2011). Anatomical variants of the biliary tract on endoscopic cholangiography [Article in Spanish]. Rev Gastroenterol Mex.

[4] Abdelsalam AM, Elansary AM, Mostafa Ibrahim Mohamed A (2021). A case report of double cystic duct during laparoscopic cholecystectomy in patient with chronic calcular cholecystitis. Int J Surg Case Rep.

[5] Salih AM, Kakamad FH, Mohammed SH, Salih RQ, Habibullah IJ, Muhialdeen AS, Fatih H (2017). Double cystic duct, a review of literature with report of a new case. Int J Surg Case Rep.

[6] Janssen BV, van Laarhoven S, Elshaer M, Cai H, Praseedom R, Wang T, Liau SS (2021). Comprehensive classification of anatomical variants of the main biliary ducts. Br J Surg.

[7] Kurahashi S, Komatsu S, Matsumura T (2020). A novel classification of aberrant right hepatic ducts ensures a critical view of safety in laparoscopic cholecystectomy. Surg Endosc.

[8] Jarnagin WR, Allen PJ, Chapman WC (2022). Blumgart's Surgery of the Liver, Biliary Tract and Pancreas, 2-Volume Set, Edition 7. https://www.inspectioncopy.elsevier.com/book/details/9780323697842.

[9] Otaibi W, Quach G, Burke B (2015). Double cystic duct in a septated gallbladder. J Investig Med High Impact Case Rep.

[10] Chrysikos D, Taprantzis N, Shihada A, Troupis T (2026). Cystic duct anatomical variations and the risk of choledocholithiasis: a systematic review and meta-analysis. Ann Gastroenterol.

